# Evolution of Skin Temperature after the Application of Compressive Forces on Tendon, Muscle and Myofascial Trigger Point

**DOI:** 10.1371/journal.pone.0129034

**Published:** 2015-06-12

**Authors:** Marina Figueiredo Magalhães, Almir Vieira Dibai-Filho, Elaine Caldeira de Oliveira Guirro, Carlos Eduardo Girasol, Alessandra Kelly de Oliveira, Fabiana Rodrigues Cancio Dias, Rinaldo Roberto de Jesus Guirro

**Affiliations:** Postgraduate Program in Rehabilitation and Functional Performance, Department of Biomechanics, Medicine, and Rehabilitation of the Locomotor Apparatus, Medical School of Ribeirão Preto, University of São Paulo, Ribeirão Preto, SP, Brazil; University of Bari, ITALY

## Abstract

Some assessment and diagnosis methods require palpation or the application of certain forces on the skin, which affects the structures beneath, we highlight the importance of defining possible influences on skin temperature as a result of this physical contact. Thus, the aim of the present study is to determine the ideal time for performing thermographic examination after palpation based on the assessment of skin temperature evolution. Randomized and crossover study carried out with 15 computer-user volunteers of both genders, between 18 and 45 years of age, who were submitted to compressive forces of 0, 1, 2 and 3 kg/cm^2^ for 30 seconds with a washout period of 48 hours using a portable digital dynamometer. Compressive forces were applied on the following spots on the dominant upper limb: myofascial trigger point in the levator scapulae, biceps brachii muscle and palmaris longus tendon. Volunteers were examined by means of infrared thermography before and after the application of compressive forces (15, 30, 45 and 60 minutes). In most comparisons made over time, a significant decrease was observed 30, 45 and 60 minutes after the application of compressive forces (p < 0.05) on the palmaris longus tendon and biceps brachii muscle. However, no difference was observed when comparing the different compressive forces (p > 0.05). In conclusion, infrared thermography can be used after assessment or diagnosis methods focused on the application of forces on tendons and muscles, provided the procedure is performed 15 minutes after contact with the skin. Regarding to the myofascial trigger point, the thermographic examination can be performed within 60 minutes after the contact with the skin.

## Introduction

Infrared thermography is a technique with health sciences applications that is used in measuring skin temperature [1,2]. It is a noninvasive and painless method that does not require any contact with the body region being assessed. It is based on the emission of infrared radiation by bodies with temperatures above absolute zero, providing an image of the thermal distribution in the body in order to measure temperature [2,3].

Many authors have investigated the evolution of skin temperature in different clinical conditions, such as temporomandibular disorder [4], neck pain [5], myofascial pain [6], diabetic neuropathy [7] and breast cancer [8]. In addition, recent clinical trials reported the use of infrared thermography before and after the application of physiotherapy resources [9–11].

In this context, it is important to note that the measurement methods and trial conditions to which individuals are submitted during thermographic assessment can have an effect on skin temperature. Therefore, Lahiri et al. [2] conducted a review study in which they found that studies that used infrared thermography had an acclimatization period of 5 to 20 minutes in an environment with temperatures varying from 18 to 25°C. According to Roy et al. [12], patients’ acclimatization time should be between 8 and 16 minutes. In addition, Costa et al. [4] highlighted the importance of having an environment without heat-generating equipment, drafts or sunlight near or on the subjects, and lighting with fluorescent lamps. Regarding the position of patients, Roy et al. [13] reported that the skin temperature of a body region does not vary according to posture during assessment.

Based on the above aspects and also considering that some assessment and diagnosis methods require palpation or the application of certain forces on the skin, which affects the structures beneath, as in the case of diagnosis of myofascial trigger points define by Simons et al. [14], one could highlight the importance of defining possible influences on skin temperature as a result of this physical contact. Thus, the objective of the present study was to assess the evolution of skin temperature after the application of compressive forces on tendon, muscle and myofascial trigger point, and determine the ideal time for performing thermographic examination after palpation. The hypothesis of this study was that an acclimatization period of 15 minutes [12] is enough to recover baseline temperature after the application of compressive forces.

## Materials and Methods

The present study was approved by the Research Ethics Committee of the Clinics Hospital (Medical School of Ribeirão Preto, University of São Paulo), under number 030643/2013. All volunteers validated their participation in the study by signing a free and informed consent form.

This is a prospective clinical study, using a crossover, blind and randomized design, in which the physiotherapist who captured and analyzed the infrared images did not know which compressive force were being applied on volunteers. Fifteen computer-user volunteers were selected from the university community of Ribeirão Preto (SP, Brazil) by verbal invitation and posters. Inclusion criteria were: subjects between 18 and 45 years of age, both genders, with a body mass index (BMI) between 20 and 25 kg/m^2^. In addition, the following exclusion criteria were adopted: volunteers who did use the computer for at least two hours a day [15], history of musculoskeletal, tendon or nervous injuries in the upper limb, history of fracture in the upper limb, use of painkillers, anti-inflammatory drugs or muscle relaxants during the previous week, presence of systemic or neuromuscular diseases, and medical diagnosis of fibromyalgia. According to eligibility criteria, there was no sample loss.

Prior to starting the experimental procedure, the volunteers were acclimated for 15 minutes at a temperature of 22° ± 2° C. Then, in crossover design, each volunteer was submitted randomly to compressive forces of 0, 1, 2 and 3 kg/cm^2^, with a washout period of 48 hours. The compressive force of 0 kg/cm^2^ corresponded to the absence of contact between the instrument and the skin. The spots on the dominant upper limb where the compressive forces were applied were: 1) active myofascial trigger point in the insertion of the levator scapulae; 2) medium point of the biceps brachii muscle; and 3) the palmaris longus tendon. The compressive force was applied for 30 seconds using a portable digital dynamometer (Instrutherm, model PTR-300, São Paulo, SP, Brazil) with a 1 cm^2^ rubber disk placed at the end.

Volunteers were assessed by means of infrared thermography before and after the application of compressive forces (15, 30, 45 and 60 minutes). The experimental procedures of the present study were conducted in a room at 22°C ± 2°C and 50% controlled air humidity, without heat-generating equipment, and without drafts or sunlight on the subjects. It is important to note that the first thermographic assessment after the application of the compressive force was done after an acclimatization period of 15 minutes, in accordance with to previous studies [12,16].

The assessment location was lit by fluorescent lamps. In addition, participants were asked to avoid the following in the two hours prior to data collection: hot baths or showers, using topical agents, creams or powders, participating in vigorous physical exercise, and ingesting stimulants such as caffeine, nicotine and chocolate [4,5].

A thermal camera (model T300, FLIR Systems, Wilsonville, OR, USA) was used, with 0.05°C precision and emissivity of 0.98. The instrument was stabilized for 10 minutes before the examination. Three infrared images were captured in sequence (with mean values being used for statistical analysis) at a 50-cm distance from the spot, so as to frame the assessed spot with the camera placed perpendicularly. During acclimatization, volunteers remained seated on a chair, trunk erect, with the forearms in supine position and lying on a table, so the infrared images were captured with orthostatic individuals. According to Roy et al. [13], changes in posture do not alter skin temperature. Besides, the skin at the studied spots was exposed, that is, without clothes or accessories that could influence local temperature.

**Fig 1:**
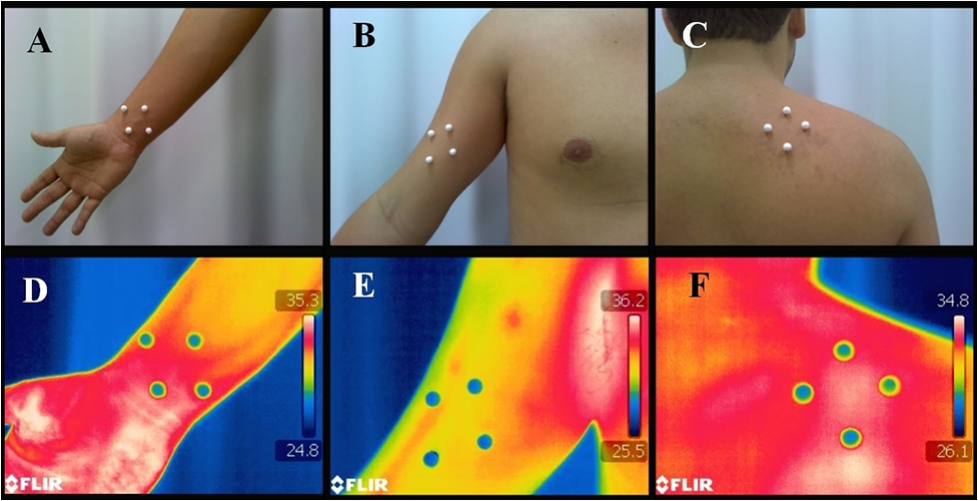
Photograph and infrared image of the palmaris longus tendon (A and D), biceps brachii muscle (B and E) and active myofascial trigger point in the insertion of the levator scapulae (C and F).

Skin temperature measurement was made by means of precise infrared image analysis, using the software QuickReport, version 1.2 (FLIR Systems). To analyze the infrared image, four 8-mm diameter Styrofoam markers were used, due to their insulating properties. They were placed at an equal distance from each other (50 mm), and the central point was the spot where the force was applied (Fig 1). A recent study pointed out excellent intra- and inter-examiner reliability for this method of analysis of infrared images, with ICC values of 0.95 and 0.90, respectively [17]. We also point out that the punctual analysis was used in this study due to the circular region of interest (extremity of the algometer in contact with skin). Furthermore, it is a well standardized method of analysis of the infrared images [17].

Regarding myofascial trigger point, it is important to note that the superior angle of the scapulae was identified, and markers were placed at equal distances in that region. After the whole experimental procedure of this study was carried out, the presence of myofascial trigger point in the predefined spots was confirmed, according to the following criteria defined by Simons et al. [14], as follows: presence of a taut band, presence of a hypersensitive point within the taut band, local contraction as a response to palpation of the taut band, and pain as a result of compression on the hypersensitive point. Myofascial trigger point was considered active when a volunteer reported spontaneous pain or previously known pain after compression [14]. This type of evaluation has high levels of inter-examiner reliability, varying from 0.84 to 0.88 [18]. The determination of myofascial trigger point was made by a physiotherapist with eight years of clinical experience in the assessment and treatment of patients with myofascial pain.

The statistical analysis was conducted by a blind researcher for the compressive forces applied. Data were presented as means, standard deviation (SD) and a confidence interval (CI) of 95%. Initially, Shapiro-Wilk test was used to evaluate data distribution. Once normality was ascertained, two-way repeated measures analysis of variance post hoc Bonferroni was used for each structure (tendon, muscle and myofascial trigger point), with two factors considered: time (before and 15, 30, 45 and 60 minutes after) and compressive force (0, 1, 2 and 3 kg/cm^2^). Mauchly’s test of sphericity with the Greenhouse-Geisser correction was considered in this analysis. A significance level of 5% was adopted for all analyses. Data were processed using SPSS, version 17.0 (Chicago, IL, USA).

## Results

Fifteen volunteers from both genders (13 women), right-handed, with an average age of 21.53 years (SD = 3.94) and mean BMI of 22.30 kg/m^2^ (SD = 1.64), met the eligibility criteria and were submitted to compressive forces of 0, 1, 2 and 3 kg/cm^2^ in crossover design and randomly.

Force-by-time interaction was not observed for the palmaris longus tendon (F_3,11_ = 0.211, p = 0.958), biceps brachii muscle (F_3,11_ = 0.887, p = 0.550) or myofascial trigger point of the levator scapulae (F_3,11_ = 0.084, p = 0.993). However, in most comparisons made over time, a significant decrease was observed 30, 45 and 60 minutes after the application of compressive forces (p < 0.05) on the palmaris longus tendon and biceps brachii muscle. Tables 1 and 2 show other details.

**Table 1:** Comparison of the skin temperature (degrees Celsius) on biceps the brachii muscle over time and at different compressive forces.

Compressive force	P0	P15	P30	P45	P60
0 kg/cm^2^	31.48 (30.93, 32.02)^a^ ^,^ ^b^ ^,^ ^c^	31.16 (30.57, 31.76)	30.93 (30.31, 31.56)	30.76 (30.17, 31.35)	30.66 (30.01, 31.32)
1 kg/cm^2^	31.57 (31.04, 32.10)^a^ ^,^ ^b^	31.43 (30.86, 32.08)^a^	31.13 (30.53, 31.73)	30.89 (30.31, 31.47)	30.76 (30.15, 31.36)
2 kg/cm^2^	31.56 (31.02, 32.10)^a^	31.75 (31.16, 32.33)^a^ ^,^ ^b^ ^,^ ^c^	31.18 (30.58, 31.78)	31.09 (30.47, 31.71)	30.83 (30.18, 31.46)
3 kg/cm^2^	31.64 (31.10, 32.18)^a^	31.86 (31.27, 32.44)^a^ ^,^ ^b^ ^,^ ^c^	31.51 (30.91, 32.11)	31.18 (30.56, 31.80)	30.89 (30.24, 31.54)

Values shown in mean (95% confidence interval). All comparisons were made by means of two-way repeated measures analysis of variance post hoc Bonferroni. P0: Before the application of compressive force; P15: Fifteen minutes after; P30: Thirty minutes after; P45: Forty-five minutes after; P60: Sixty minutes after.

^a^Differs significantly from P60 (p < 0.05)

^b^Differs significantly from P45 (p < 0.05)

^c^Differs significantly from P30 (p < 0.05).

**Table 2:** Comparison of the skin temperature (in degrees Celsius) on the palmaris longus tendon over time and at different compressive forces.

Compressive force	P0	P15	P30	P45	P60
0 kg/cm^2^	31.81 (31.12, 32.50)^a^	31.80 (30.89, 32.71)^a^ ^,^ ^b^	31.39 (30.39, 32.39)^a^ ^,^ ^b^	30.82 (29.82, 31.81)^a^	30.32 (29.31, 31.32)
1 kg/cm^2^	31.73 (30.73, 32.73)^a^	31.68 (30.97, 32.40)^a^ ^,^ ^b^	31.26 (30.52, 32.02)^a^ ^,^ ^b^	30.75 (29.91, 31.58)^a^	30.35 (29.50, 31.20)
2 kg/cm^2^	31.50 (30.50, 32.50)	31.61 (30.89, 32.32)^a^ ^,^ ^b^	31.32 (30.57, 32.07)^a^ ^,^ ^b^	30.81 (29.97, 31.64)^a^	30.35 (29.50, 31.19)
3 kg/cm^2^	32.20 (31.22, 33.22)^a^ ^,^ ^b^	32.05 (31.33, 32.76)^a^ ^,^ ^b^ ^,^ ^c^	31.56 (30.81, 32.31)^a^ ^,^ ^b^	31.01 (30.18, 31.85)^a^	30.59 (29.75, 31.44)

Values shown in mean (95% confidence interval). All comparisons were made by means of two-way repeated measures analysis of variance post hoc Bonferroni. P0: Before the application of compressive force; P15: Fifteen minutes after; P30: Thirty minutes after; P45: Forty-five minutes after; P60: Sixty minutes after.

^a^Differs significantly from P60 (p < 0.05)

^b^Differs significantly from P45 (p < 0.05)

^c^Differs significantly from P30 (p < 0.05).

Table 3 displays values of skin temperature of the levator scapulae with active myofascial trigger point. Therefore, no significant differences were observed over time or between compressive forces applied (p > 0.05).

**Table 3:** Comparison of the skin temperature (in degrees Celsius) on myofascial trigger point over time and at different compressive forces.

Compressive force	P0	P15	P30	P45	P60
0 kg/cm^2^	33.22 (32.55, 33.89)	33.08 (32.37, 33.80)	32.88 (32.15, 33.62)	32.77 (31.98, 33.56)	32.68 (31.88, 33.48)
1 kg/cm^2^	33.27 (32.32, 34.23)	33.17 (32.58, 33.75)	32.95 (32.35, 33.55)	32.85 (32.25, 33.46)	32.78 (32.16, 33.40)
2 kg/cm^2^	33.93 (32.98, 34.89)	33.89 (33.11, 34.48)	33.57 (32.97, 34.17)	33.50 (32.90, 34.10)	33.50 (32.89, 34.12)
3 kg/cm^2^	33.71 (32.76, 34.67)	33.47 (32.89, 34.06)	33.38 (32.78, 33.98)	33.31 (32.71, 33.92)	33.27 (32.65, 33.88)

Values shown in mean (95% confidence interval). All comparisons were made by means of two-way repeated measures analysis of variance post hoc Bonferroni. P0: Before the application of compressive force; P15: Fifteen minutes after; P30: Thirty minutes after; P45: Forty-five minutes after; P60: Sixty minutes after.

No significant differences in comparisons over time or between different compressive forces (p > 0.05).

## Discussion

In the present study, in most comparisons made over time, it was observed a decrease in skin temperature on the palmaris longus tendon and biceps brachii muscle 30, 45 and 60 minutes after the application of compressive forces. For myofascial trigger point, no changes in skin temperature were observed after the application of compressive forces.

Infrared thermography is a simple method that is easy to apply for measuring skin temperature [2, 3]. However, reliable figures that correspond to skin temperature depend on the equipment used [2], the analysis method for the infrared images [4], and the experimental conditions in which the examination is performed, such as room temperature, time of acclimatization and intake of thermogenic substances [4,12,19,20]. In this context, the present study stands out in the literature for aiming to identify possible influences of compressive forces on skin temperature.

One clinical study used a mechanical force with manual assistance in healthy subjects and observed a decrease in skin temperature immediately after the application of a force on the lumbar region, with subsequent heating [21]. The authors explained that the flow decrease was related to the mechanical deformation of the skin with the application of the force, and the increase of secondary blood flow was related to reflex capillary dilatation, followed by the increase of capillary and venule permeability. In present study, in general, a decrease in temperature was observed in the biceps brachii muscle and the palmaris longus tendon 30, 45 and 60 minutes after the application of compressive forces (0, 1, 2 e 3 kg/cm^2^). This result can be associated with the length of exposure to an environment with a controlled temperature of 22°C ± 2°C.

According to Roy et al. [12], acclimatization times in which skin temperature is stable varies from 8 to 16 and close to the 30 minutes, being performed the evaluations in the cervical and lumbar regions. Out of those time slots, there is greater fluctuation and dispersion of skin temperature. In the present study, when considering the biceps brachii muscle, our results are partially concordant to the study conducted by Roy et al. [12] to the not observe significant difference in the comparisons performed in the following time periods: 0 and 15 minutes, 30 and 45 minutes, 45 and 60 minutes, and 30 and 60 minutes. These findings demonstrate stability of skin temperature in these periods of time, as shown Table 1. However, when considering the palmaris longus tendon, it was not observed the stabilization of temperature during the period of time between 30 and 60 minutes, as shown in Table 2.

Thus, when evaluated over time and in acclimatized environment, the skin temperature on different structures (muscle belly, tendon and myofascial trigger point) has different behaviors. Complementarily, to advance the understanding of the skin temperature behavior over time, we suggest the conducting of future studies searching for relationships between skin and core temperature for at least 60 minutes, since the study conducted by Roy et al. [12] evaluated the skin temperature for 30 minutes.

There were no changes in skin temperature on the active myofascial trigger point after the application of different compressive forces. A possible reason for this is associated with the fact that myofascial trigger points are pathological structures found in dysfunctional muscles that trigger motor, sensitive and autonomic changes [22], besides provoking metabolic changes with several algesic substances in the region [23]. However, the present study does not provide specific methodology to test this hypothesis.

Palpation is part of physical examination and is commonly used by physiotherapists in clinical environment, as reported by several studies. Park et al. [24] performed palpation to identify pathological changes in the temporomandibular joint. In the event of traumatic injury, Miller et al. [25] performed palpation on the calcaneal tendon and medial malleolus as part of the investigation of fractures. A recent systematic review highlights palpation, associated with other techniques, as a common method used for diagnosis of hamstring injuries [26]. In addition to these clinical conditions, palpation is the most widely accepted diagnostic procedure for identifying myofascial trigger points [14,27].

In the present study, a 30-second compression was performed with loads of 1, 2 and 3 kg/cm^2^ as a means of mimicking different forces applied in palpation performed in clinical practice, and the possibility of using infrared thermography after 15 minutes of compression was found. Thus, palpation and infrared thermography can be combined for the assessment of clinical conditions, especially in the investigation of painful experiences [11,16,28,29].

This study had some limitations. The application of forces and the measurement of skin temperature were not performed in other muscle groups, tendons or body regions (face, chest, abdomen, back and lower limbs), since there are variations in baseline temperature patterns according to body region [20], and latent myofascial trigger points were not evaluated. Finally, we suggest that future studies use infrared thermography and palpation in other pathological conditions, as long as their combination is clinically applicable.

## Conclusion

Infrared thermography can be used after assessment or diagnosis methods focused on the application of forces on tendons and muscles, provided the procedure is performed 15 minutes after contact with the skin. Regarding to the myofascial trigger point, the thermographic examination can be performed within 60 minutes after the contact with the skin.
